# Low-Temperature Synthesis of Monetite/Dextran Hybrid Coatings with Tailored Corrosion Resistance on Ti6Al4V

**DOI:** 10.1021/acsomega.5c05440

**Published:** 2025-08-18

**Authors:** Robert S. Matos, Henrique D. da Fonseca Filho, Michael D. S. Monteiro, Nilson S. Ferreira

**Affiliations:** † Amazonian Materials Group, Federal University of Amapá, Macapá 68903-419, AP, Brazil; ‡ Laboratory for Development and Applications of Amazon Nanomaterials (LADENA), Department of Materials Physics, 67892 Federal University of Amazonas, Manaus 69067-005, AM, Brazil; § Department of Physics, 74391 Federal University of Sergipe, 49100-000 São Cristovão, SE, Brazil

## Abstract

Hybrid coatings composed of crystalline monetite (CaHPO_4_) and kefir-derived Dextran were synthesized on Ti6Al4V substrates using a low-temperature sol–gel-assisted route (≤80 °C), enabling biopolymer integration without thermal degradation. X-ray diffraction (XRD) confirmed the formation of triclinic monetite nanocrystals (∼152 nm), while Fourier transform infrared (FTIR) and scanning electron microscopy/energy dispersive X-ray spectroscopy (SEM/EDS) analyses verified the uniform incorporation of Dextran, particularly in the 4 wt % formulation, which yielded compact, homogeneous surfaces. Electrochemical evaluations in Fusayama artificial saliva revealed a substantial enhancement in corrosion resistance, with the open-circuit potential shifting from −0.272 V (uncoated) to −0.034 V and polarization resistance increasing from 1987.6 to 5573.4 Ω. This performance surpasses several benchmark coatings reported for biomedical alloys, highlighting the synergistic interaction between the inorganic phase and the biopolymeric matrix. The hybrid architecture promotes defect sealing, barrier formation, and interface stabilization. These results position the monetite/Dextran system as a scalable, sustainable, and biocompatible solution for next-generation implant coatings, offering a mild-temperature alternative to traditional ceramic deposition methods.

## Graphical abstract

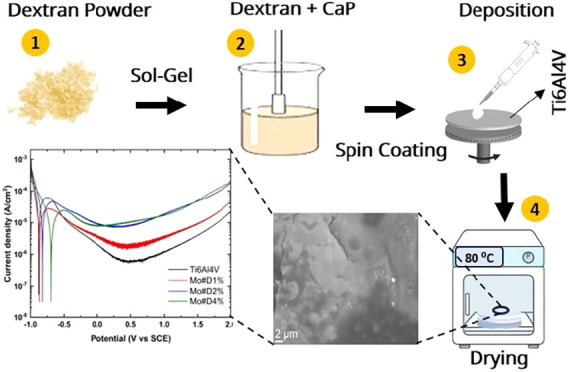

## 1 Introduction

Titanium alloys are widely used in biomedical implants due to their favorable mechanical properties and biocompatibility.^1^ However, their long-term performance can be compromised by limited corrosion resistance in physiological environments. To overcome this challenge, surface coatings that combine protective and bioactive functions have gained increasing attention. Recent advances in surface functionalization have focused on enhancing the bioactivity, corrosion resistance, and osseointegration of titanium-based implants, particularly Ti6Al4V, which remains a gold standard in orthopedic and dental fields due to its excellent mechanical strength and biocompatibility. Despite these advantages, its native oxide layer offers limited bioactivity, often necessitating the development of surface coatings capable of promoting cellular interaction while protecting against degradation in physiological environments.^1,2^ A wide range of strategies has been proposed to address this limitation, including plasma electrolytic oxidation (PEO), microarc oxidation (MAO), and sol–gel-assisted deposition methods. For instance, Nadaraia et al.^3^ reported the formation of composite PEO–PDA coatings on three-dimensional (3D)-printed titanium, incorporating hydroxyapatite and polydopamine to improve corrosion resistance and biomimetic mineralization. Similarly, Coan et al.^4^ developed MAO coatings doped with transition metal oxides (TiO_2_, MoO_3_, Fe_2_O_3_, and MnO_2_) and bioactive Ca/P species, achieving enhanced wear resistance, osteogenic potential, and antimicrobial selectivity.

Beyond electrochemical methods, sol–gel and biomimetic routes have gained attention for their ability to process hybrid organic–inorganic systems at mild temperatures.^5^ Ansari et al.^6^ developed sol–gel chitosan/calcium phosphate hybrid coatings on Ti6Al4V via sol–gel, achieving uniform, crack-free surfaces with improved wettability and antibacterial activity against *Staphylococcus aureus* and *Escherichia coli*, suitable for implant applications. Likewise, Jha et al.^7^ fabricated multifunctional sol–gel coatings combining chitosan and calcium phosphate on Ti6Al4V, achieving dense, crack-free surfaces with enhanced corrosion resistance, antibacterial performance, and bioactivity suitable for orthopedic implant applications. In this regard, biopolymers such as kefiran, Dextran, and pullulan stand out due to their film-forming capacity, hydrophilicity, and biodegradability.^8^ However, their thermal sensitivity remains a critical challenge when integrating with conventional high-temperature ceramic processing. Recently, Lukaviciute et al.^9^ highlighted the advantages of low-temperature coating strategies in preserving the functional integrity of biopolymeric components while promoting synergistic effects in hybrid film architectures.

Among bioactive materials, monetite (CaHPO_4_), a metastable calcium phosphate phase, is notable for its osteoconductivity, resorbability, and compatibility with bone tissue.^10,11^ Furthermore, polysaccharides such as Dextran offer excellent biocompatibility, film-forming properties, and potential to improve interfacial adhesion in composite coatings.^12^ Despite notable advances in the development of calcium phosphate-based coatings, most fabrication techniques still rely on high-temperature processing steps,^13^ which are incompatible with thermally labile components such as biopolymers. This thermal incompatibility poses a significant challenge for the design of hybrid systems, as elevated temperatures can compromise the structural integrity and functional properties of organic constituents like Dextran.^2^ In particular, the integration of Dextran, especially kefir-derived Dextran, into Monetite (CaHPO_4_)-based coatings remains largely unexplored in the current literature. Moreover, there is a critical gap in systematic studies evaluating how varying concentrations of Dextran influence the structural, morphological, and electrochemical characteristics of such hybrid coatings when synthesized under low-temperature or sol–gel-assisted mild conditions. Addressing this gap is essential for optimizing coating performance and expanding their applicability in biomedical environments.

Herein, we present hybrid Monetite/Dextran coatings developed on Ti6Al4V substrates using a low-temperature sol–gel-assisted method. The coatings were thoroughly characterized, and corrosion testing confirmed their enhanced protective performance. The process, carried out at ≤80 °C, preserved the structural integrity of the Dextran component. The approach ensures molecular-level dispersion of kefir-derived Dextran without compromising its structural integrity, enabling uniform coverage and enhanced corrosion resistance. The proposed method complements existing techniques by offering a sustainable and biocompatible alternative, with potential applications in next-generation biomedical implants. This eco-friendly approach offers a versatile route to biobased, multifunctional coatings with potential for biomedical and surface protection applications.

## 2 Materials and Methods

### 2.1 Synthesis

Dextran was isolated following a reported protocol.^14^ Monetite/Dextran composites were synthesized via a modified sol–gel method. A calcium phosphate suspension was prepared using calcium nitrate tetrahydrate (Ca­(NO_3_)_2_·4H_2_O, Sigma-Aldrich, 0.167 mol/L, >98%), Ammonium hydrogen phosphate ((NH_4_)_2_HPO_4_, Sigma-Aldrich, 0.1 mol/L, >98%), and Urea ((NH_2_)_2_CO, Sigma-Aldrich, 0.5 mol/L, >98%), with pH adjusted to 3. Dextran (1, 2, and 4% w/v) was dissolved in ultrapure water at 85 °C and mixed with the CaP suspension (1.5:1). The mixture was autoclaved at 80 °C for 3 h and then incubated in a water bath at the same temperature. Ti6Al4V discs (2 cm^2^) were coated with 10 layers of the composite via spin coating at 2000 rpm with 1 min intervals, followed by drying at 70 °C for 2 h.

### 2.2 Characterization

Thermogravimetric analysis (TGA) was performed using a Shimadzu DTG-60H from room temperature to 400 °C at 10 °C/min under N_2_. Attenuated total reflectance-Fourier transform infrared (ATR-FTIR) spectra were acquired on an Agilent Cary 630 in the 650–4000 4 cm^–1^ range at 4 cm^–1^ resolution. X-ray diffraction (XRD) was conducted using a Shimadzu LabX XRD-6000 (Cu Kα, λ = 1.54056 Å, 40 kV, 40 mA, 2°/min, 2θ = 10–60°). Surface morphology and elemental composition were analyzed by scanning electron microscopy (SEM) (JEOL JSM-5700) with energy dispersive X-ray spectroscopy (EDS) at 10 kV. Corrosion resistance was evaluated in Fusayama artificial saliva using a Metrohm Autolab PGSTAT204 potentiostat. A three-electrode setup was used (SCE reference, Pt counter, coated disc working electrode). Open-circuit potentials were monitored for 50 min, followed by polarization from −1 V (vs OCP) to 2 V (vs SCE) at 0.1667 mV/s.

## 3 Results and Discussion

The thermal stability of kefir-derived Dextran was evaluated by thermogravimetric analysis (TGA) to determine appropriate postdeposition conditions for preserving the biopolymeric structure in the hybrid coating (Figure 1a). The TG curve revealed a characteristic three-step degradation profile. The first stage exhibited an initial weight loss of approximately 26% between 25–150 °C (*T*_onset_ ≈ 95 °C), which is attributed to the desorption of physically adsorbed moisture and residual solvent from the hydrophilic polysaccharide matrix. This step reflects the inherent hygroscopicity of Dextran due to the abundance of hydroxyl groups capable of hydrogen bonding with water molecules.^14^ A thermal plateau was observed between 150–200 °C, indicating a relatively stable intermediate region with minimal mass change. This plateau precedes the main decomposition event and is often associated with the onset of thermal rearrangements or limited cross-linking that do not result in significant volatilization. The most prominent degradation phase occurred between 200–400 °C (*T*_onset_ ≈ 286 °C), where a major weight loss of ∼74% was recorded. This region corresponds to the thermal decomposition of the polysaccharide chains, primarily involving the cleavage of glycosidic bonds and depolymerization of the Dextran backbone. The degradation pathway leads to the release of volatile compounds such as water vapor, carbon dioxide, carbon monoxide, and low molecular weight carbonaceous fragments.^15^ The breakdown of the polymer structure in this range is consistent with the known thermal behavior of Dextran and similar microbial exopolysaccharides such as kefiran,^14^ which exhibit a sharp decline in mass due to the scission of C–O–C linkages and subsequent volatilization of degradation products. Based on this thermal profile, a postdeposition heat treatment at 70 °C was selected to ensure efficient solvent evaporation while remaining well below the decomposition onset, thereby preserving the structural and functional integrity of the kefir-derived Dextran component in the final hybrid coating.

**Figure 1:**
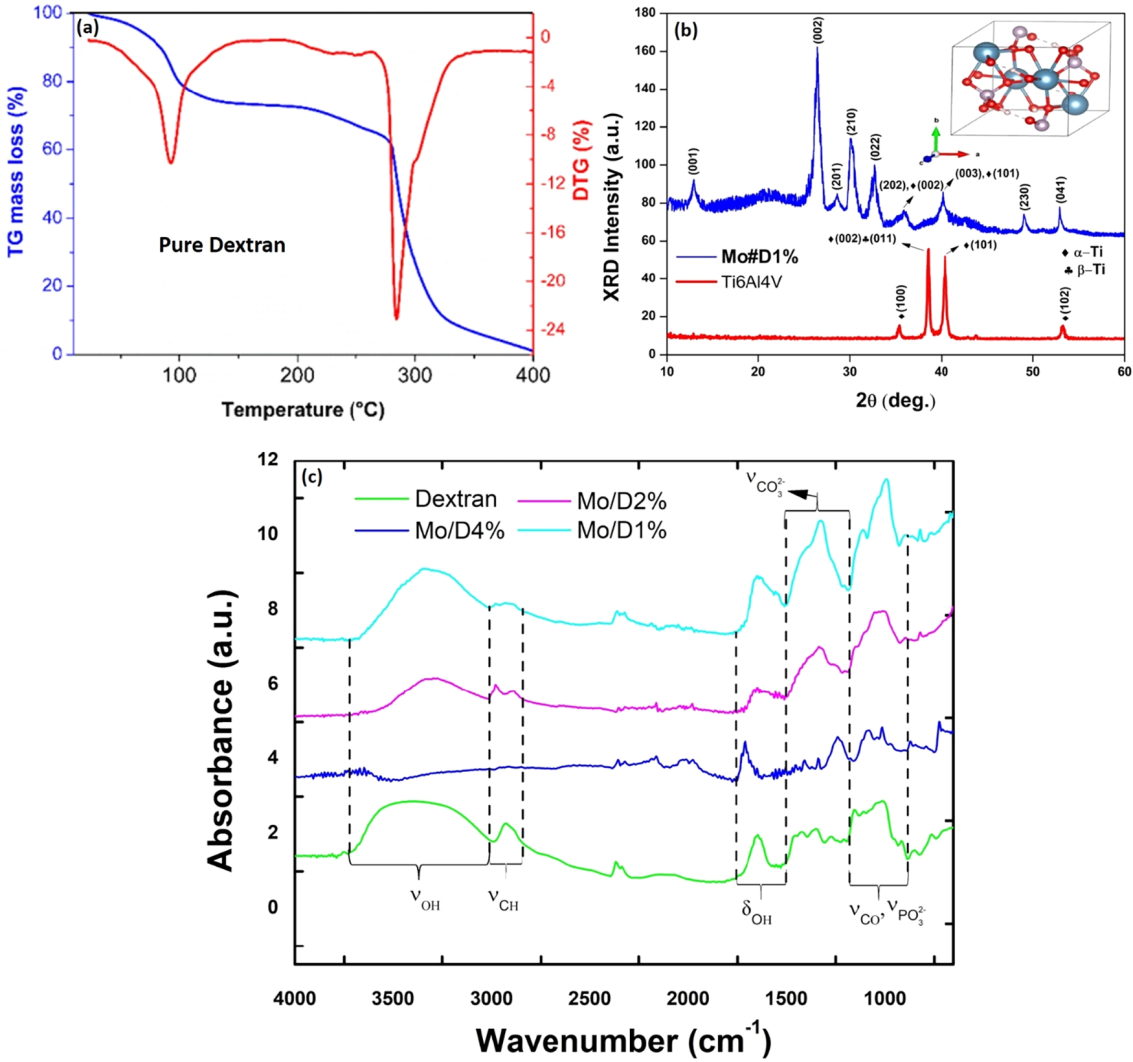
(a) TG/DTG curves of the pure Dextran biopolymer. (b) XRD pattern of the Monetite coating loaded with 1% Dextran; the inset shows the crystal structure of Monetite. (c) FTIR spectra of pure Dextran and Monetite/Dextran composites.

The XRD pattern of the Monetite/Dextran composite containing 1 wt % Dextran (Figure 1b) displays well-defined diffraction peaks characteristic of crystalline CaHPO_4_, confirming successful formation of the Monetite phase. CaHPO_4_ crystallizes in a triclinic unit cell with an anorthic structure (space group *P*1, ICSD#87196), and the observed reflections, indexed to the (001), (002), (210), (201), (022), (202), (003), (230), and (041) planes, are in good agreement with the reference pattern. The average crystallite size, estimated using the Scherrer equation,^16^ is approximately 152 nm, indicative of a well-developed crystalline phase. For comparison, the XRD pattern of the bare Ti6Al4V substrate is included in Figure 1b, showing characteristic diffraction peaks of both α–Ti (space group *P*63/*mmc*, ICSD#076265) and β–Ti (space group *Im*3̅*m*, ICSD#076165) phases. The absence of Monetite-specific reflections in the uncoated alloy unequivocally confirms that the crystalline CaHPO_4_ phase is formed exclusively as a result of the coating process. Additionally, a broad halo centered between 15–20° (2θ) suggests the presence of an amorphous component, likely associated with the semicrystalline nature of Dextran. This diffuse scattering does not overlap with the principal diffraction peaks, implying molecular-level dispersion or partial disorder of the polymer within the matrix. Collectively, the data support the formation of a hybrid structure comprising a crystalline Monetite phase embedded in an amorphous Dextran-rich environment.

FTIR analysis further supported this hybrid nature (Figure 1c). The spectrum of pure kefir-derived Dextran displayed typical polysaccharide bands: a broad O–H stretching vibration at 3420 cm^–1^, C–H stretching at 2929 cm^–1^, and H–O–H bending at 1673 cm^–1^. Additional bands at 1360 cm^–1^ (CH_2_/O–H bending), 1000–1200 cm^–1^ (C–O–C stretching), and 900–530 cm^–1^ (α-d-glycosidic linkages) confirm the carbohydrate backbone.^17^ The weak band at 2359 cm^–1^ is attributed to the asymmetric stretching of trace atmospheric CO_2_ physically adsorbed onto the hydrophilic Dextran matrix during sample preparation or FTIR data acquisition, and is not related to any structural or decomposition-derived species. In the composites, new bands appeared at ∼962, 1030–1090, and a doublet at ∼602 and 563 cm^–1^, corresponding to of PO_4_^3–^ vibrations, validating the formation of Monetite. Notably, shifts and intensity changes in the fingerprint region with increasing Dextran content suggest specific molecular interactions, likely hydrogen bonding, between the phosphate groups and the polysaccharide chains, in agreement with the hybrid structure observed by XRD. These structural and chemical modifications were accompanied by clear morphological evolution, as observed in SEM images (Figure 2a–c), which show a progressive surface transition from granular textures at low Dextran content (Mo#D1%) to smoother, more homogeneous coatings at higher concentrations (Mo#D4%). This transformation suggests polymer-induced matrix restructuring during hybrid formation. EDS spectra (Figure 2d–e) further support this trend, revealing a marked reduction in calcium and phosphorus signals, complete masking of the Ti6Al4V substrate, and a concurrent increase in carbon and oxygen content, evidence of increasing organic phase incorporation. At 4 wt % Dextran, the coating appears fully continuous and chemically organic-rich, consistent with surface homogenization and phosphate signal suppression. These observations align with FTIR data, which showed enhanced C–O–C and O–H stretching vibrations and diminished phosphate bands, confirming polymer integration and partial suppression of the inorganic phase. Altogether, these results demonstrate that Dextran content serves as a key parameter for tailoring coating morphology, composition, and hybrid character.

**Figure 2:**
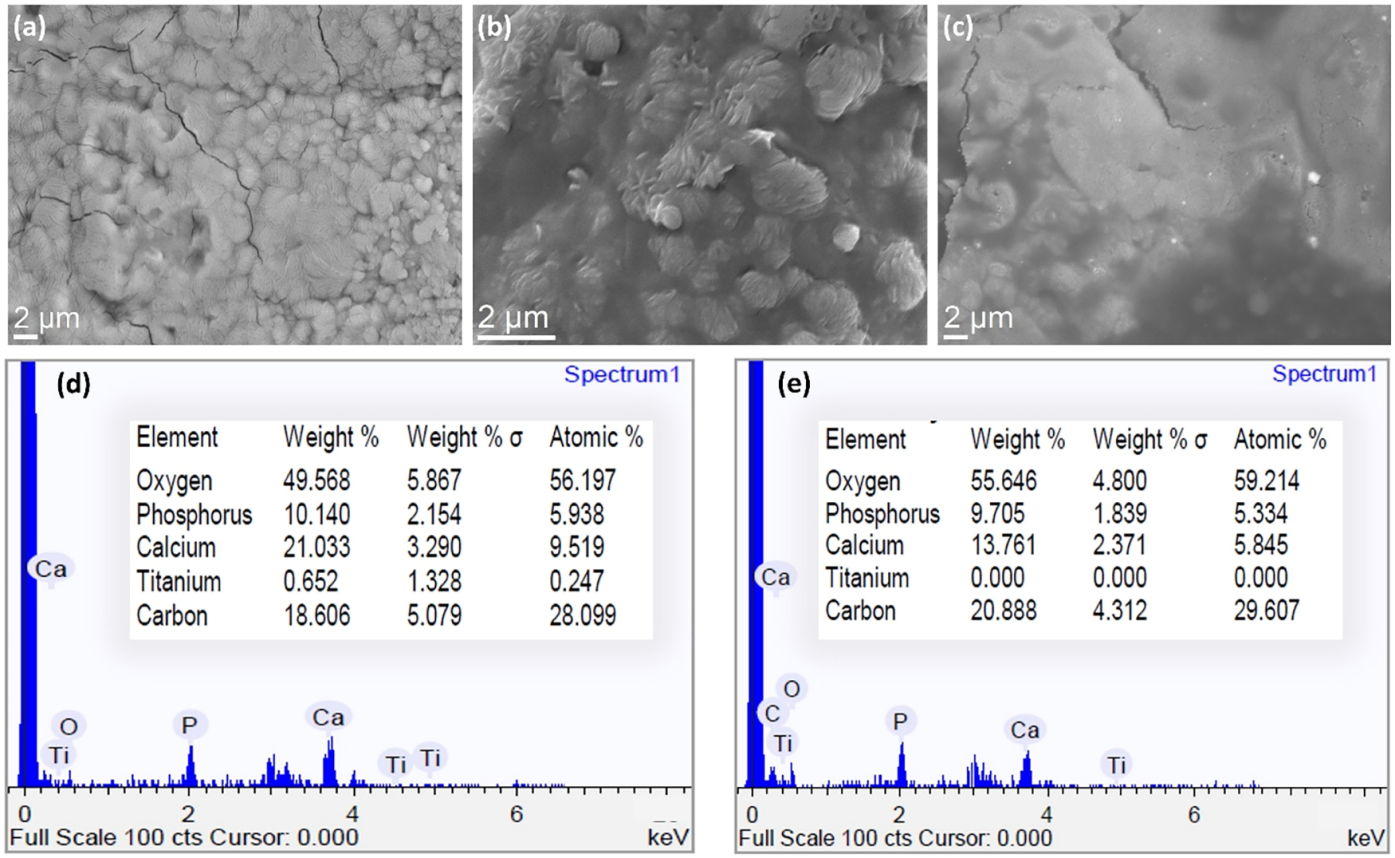
SEM micrographs of Monetite/Dextran composite coatings with increasing Dextran content: (a) Mo#D1%, (b) Mo#D2%, and (c) Mo#D4%. EDS spectra and elemental compositions are shown for (d) Mo#D1% and (e) Mo#D4%, based on mapping analysis.

Potentiodynamic polarization analysis (Figure 3 and Table 1) confirmed that the incorporation of monetite and Dextran into hybrid composite coatings significantly enhances the corrosion resistance of Ti6Al4V in simulated physiological media. The uncoated alloy exhibited the most negative open-circuit potential (OCP = −0.272 V), a corrosion potential (*E*_corr_) of −0.866, and the highest corrosion current density (*I*_corr_ = 1.799 × 10^–5^ A/cm^2^), all characteristic of an unstable passive layer and active metal dissolution. Conversely, coatings containing increasing concentrations of Dextran, especially the Mo#D4% formulation, induced a marked shift in OCP toward more noble values (−0.034 V) and reduced *I*_corr_ to 1.074 × 10^–5^ A/cm^2^, while elevating the polarization resistance (*R*_p_) to 5573.4 Ω (Table 1), more than twice that of the bare substrate. This improved electrochemical behavior indicates the formation of a more robust and protective passive film. The enhancement is attributed to the synergistic interplay between the crystalline monetite, which provides a dense, inorganic barrier to electrolyte penetration, and the biopolymeric Dextran matrix, which improves coating integrity, seals microdefects, and likely reduces water and ion transport pathways. Although the Mo#D2% sample exhibited the lowest *I*_corr_ value (0.858 × 10^–5^ A/cm^2^), its *R*_p_ was slightly inferior to that of Mo#D1%, possibly due to microstructural inhomogeneities that introduced localized diffusion pathways, compromising overall impedance. Our SEM analysis (Figure 2) confirms that increasing Dextran content smooths surface morphology, reduces microporosity, and limits electrolyte ingress. These structural and functional refinements collectively enhance corrosion resistance, positioning the monetite–Dextran hybrid coating as a promising solution for long-term protection of Ti6Al4V implants in biomedical applications.

**Figure 3:**
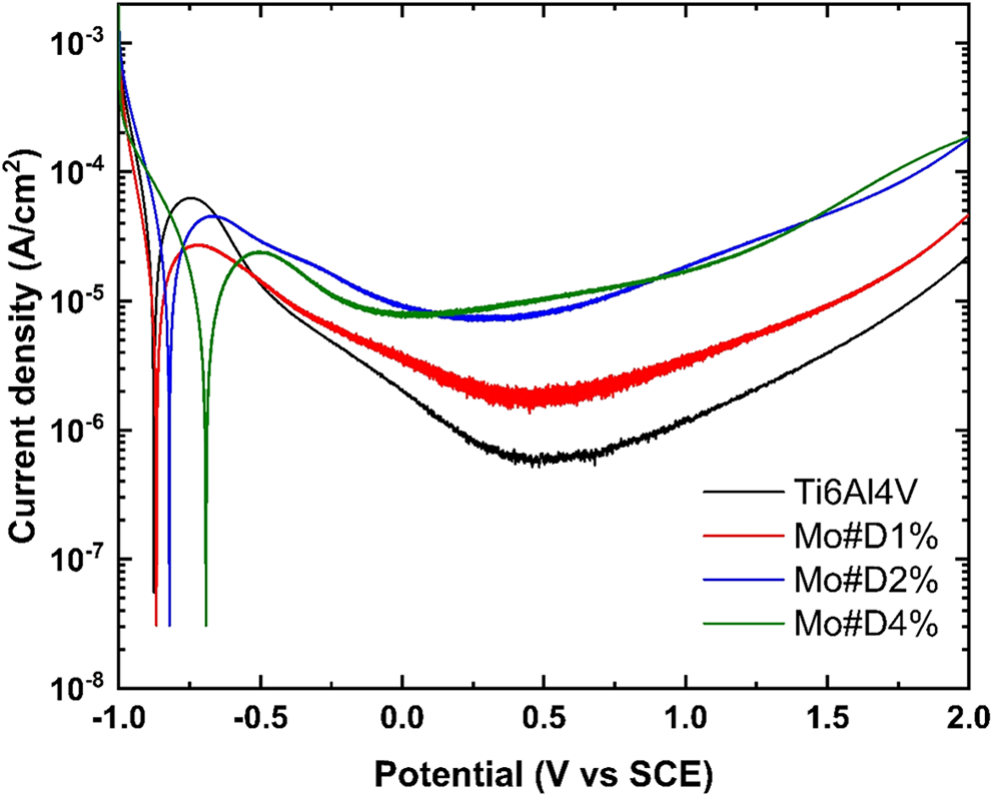
Potentiodynamic polarization curves of uncoated Ti6Al4V and Monetite/Dextran-coated samples with varying Dextran content: 1% (Mo#D1%), 2% (Mo#D2%), and 4% (Mo#D4%).

**Table 1:** Open-Circuit Potential (OCP), Corrosion Potential (*E*_corr_), Corrosion Current Density (*I*_corr_), and Polarization Resistance (*R*_p_) Values of Uncoated Ti6Al4V and Monetite/Dextran-Coated Samples in Fusayama Artificial Saliva

sample	OCP (V)	*E*_corr_ (V)	*I*_corr_ (× 10^–5^ A/cm^2^)	*R*_p_ (Ω)
Ti6Al4V	–0.272	–0.866	1.799	1987.6
Mo#D1%	–0.226	–0.864	1.212	2145.9
Mo#D2%	–0.157	–0.824	0.858	1865.5
Mo#D4%	–0.034	–0.688	1.074	5573.4

Therefore, the Mo#D4% sample exhibited the most noble open-circuit and corrosion potentials, confirming its electrochemical robustness in Fusayama artificial saliva. Critically, these results surpass several state-of-the-art coating systems reported in the existing literature. For instance, Hussein and Fekry^18^ described a fumed silica/chitosan/PVP coating with excellent impedance (17.9 MΩ·cm^2^), but their synthesis involved multicomponent chemistries and high material cost, in contrast to our bioderived, scalable, and low-temperature (<80 °C) process. Similarly, Madej et al.^19^ reported diamond-like carbon (DLC) coatings that reduced Icorr by 85% on Ti6Al4V in saliva; however, their deposition required PACVD at ∼220 °C under vacuum, conditions incompatible with biopolymer integrity and widespread biomedical adoption. Moreover, Ciobotaru and Banu^20^ assessed uncoated Ti6Al4V under identical electrolyte conditions and found corrosion current densities around 1.90 μA/cm^2^, notably higher than our Mo#D4% formulation, which achieved 0.1074 μA/cm^2^ without pH buffering. Further, Zheng et al.^21^ employed hot isostatic pressing on electron beam powder bed fused Ti6Al4V, achieving an *I*_corr_ of 2.45 × 10^–5^ A/cm^2^ and a *R*_p_ of ∼2150 Ω. Zhang et al.,^22^ on the other hand, studied Ti6Al4V fabricated via selective laser melting and subjected to artificial saliva containing fluoride at acidic pH, reporting an even higher *I*_corr_ of 3.32 × 10^–5^ A/cm^2^ and a lower *R*_p_ of ∼1380 Ω. Despite the sophisticated processing routes employed in both studies, the corrosion resistance of these materials remains inferior to that of our Mo#D4% monetite–Dextran coating, which exhibited a significantly lower Icorr and a markedly higher *R*_p_ values under similar simulated physiological conditions. This enhanced protection results from the synergistic barrier effect of monetite and Dextran, which together resist electrolyte diffusion and improve stability, making the coating a promising alternative for protecting Ti6Al4V implants in biomedical environments.

## 4 Conclusions

In conclusion, we have developed a low-temperature, sol–gel-derived hybrid coating comprising crystalline Monetite and kefir-derived Dextran, applied to Ti6Al4V substrates. Structural and spectroscopic analyses confirmed a biphasic architecture, while morphological and electrochemical data demonstrated that increasing Dextran content improves surface uniformity and corrosion resistance. The Mo#D4% formulation achieved over 2-fold enhancement in polarization resistance under simulated physiological conditions. These results underscore the potential of biopolymer-assisted hybrid coatings as sustainable, cost-effective alternatives for the protection of titanium-based biomedical devices.
